# Eggshell Spottiness Reflects Maternally Transferred Antibodies in Blue Tits

**DOI:** 10.1371/journal.pone.0050389

**Published:** 2012-11-30

**Authors:** Marie-Jeanne Holveck, Arnaud Grégoire, Vincent Staszewski, Romain Guerreiro, Philippe Perret, Thierry Boulinier, Claire Doutrelant

**Affiliations:** Ecologie Evolutive, Centre d'Ecologie Fonctionnelle et Evolutive (CEFE-CNRS), Montpellier, France; University of Bern, Switzerland

## Abstract

Blue-green and brown-spotted eggshells in birds have been proposed as sexual signals of female physiological condition and egg quality, reflecting maternal investment in the egg. Testing this hypothesis requires linking eggshell coloration to egg content, which is lacking for brown protoporphyrin-based pigmentation. As protoporphyrins can induce oxidative stress, and a large amount in eggshells should indicate either high female and egg quality if it reflects the female's high oxidative tolerance, or conversely poor quality if it reflects female physiological stress. Different studies supported either predictions but are difficult to compare given the methodological differences in eggshell-spottiness measurements. Using the blue tit *Cyanistes caeruleus* as a model species, we aimed at disentangling both predictions in testing if brown-spotted eggshell could reflect the quality of maternal investment in antibodies and carotenoids in the egg, and at improving between-study comparisons in correlating several common measurements of eggshell coloration (spectral and digital measures, spotted surface, pigmentation indices). We found that these color variables were weakly correlated highlighting the need for comparable quantitative measurements between studies and for multivariate regressions incorporating several eggshell-color characteristics. When evaluating the potential signaling function of brown-spotted eggshells, we thus searched for the brown eggshell-color variables that best predicted the maternal transfer of antibodies and carotenoids to egg yolks. We also tested the effects of several parental traits and breeding parameters potentially affecting this transfer. While eggshell coloration did not relate to yolk carotenoids, the eggs with larger and less evenly-distributed spots had higher antibody concentrations, suggesting that both the quantity and distribution of brown pigments reflected the transfer of maternal immune compounds in egg yolks. As yolk antibody concentrations were also positively related to key proxies of maternal quality (egg volume, number, yellow feather brightness, tarsus length), eggshells with larger spots concentrated at their broad pole may indicate higher-quality eggs.

## Introduction

Blue-green and red-brown eggshells have been hypothesized to be sexually selected in bird species with biparental care (i.e. the sexually selected egg color hypothesis SSECH [1], see [2], [3] for reviews and critical discussions on the hypothesis). Biliverdin is the pigment responsible for blue-green coloration and protoporphyrins result in the brown coloration of eggshell maculae or spottiness [4]. As both pigments are also involved in oxidative stress regulation in the laying female [5], their deposition in eggshells may reflect female physiological condition (e.g. antioxidant capacity) and health at the time of egg laying. For instance, a trade-off between eggshell blue-green intensity and female plasma antioxidant levels has been suggested to arise under stressful environmental conditions in pied flycatchers *Ficedula hypoleuca* [6]. A negative correlation between the levels of brown eggshell pigmentation and female immunity has also been found in blue tits *Cyanistes caeruleus* [7]. In great tits *Parus major*, the deposition of protoporphyrin pigments, which mainly derived from the blood [8], varies with female's anemic condition, which changes over laying sequence [9].

As female physiological condition can directly affect maternal investment in eggs (e.g. yolk antibodies and carotenoids [10]), eggshell color may also reflect egg and offspring quality. During the laying period, female metabolism is high, inducing high oxidative stress [11] and, as a consequence, a significant demand for antioxidants involved in somatic maintenance. It is likely that the females that most successfully trade-off between their own antioxidant defense and the color intensity of their eggs may also be more successful in transmitting antioxidants to their nestlings (although the nature of mother-egg relationships can be complex, e.g. [12]). In agreement with this prediction, avian eggs with more intense blue-green pigmentation have higher levels of carotenoids (e.g. [13], [14]). Carotenoids are antioxidants obtained exclusively from the diet, which protect molecules (including antibodies) from oxidative damage ([11], but see [15]) and play an important role in immune system stimulation and embryonic development (e.g. [16], [17], [18]). Maternally-derived yolk antibodies are another class of molecules that are essential to protecting infection-vulnerable neonates (which have not yet developed a functional immune system [19]), but their relationship with eggshell blue-green intensity varies (e.g. positive in [20], absent in [14]). While eggshell blue-green pigmentation may reflect some maternal investment in the egg, this possibility remains to be tested for brown protoporphyrin-based pigmentation.

In addition to be potentially under sexual selection pressure [1], protoporphyrins' deposition upon and/or within the eggshell during egg formation [4] may have a structural function (which does not preclude a signaling function) [21], [22]: protoporphyrins may compensate for localized eggshell thinning (e.g. caused by calcium deficiency), thereby strengthening the eggshell and reducing permeability and water loss during incubation (reviewed in [3]). However, as expected for calcium-supplemented birds, a decrease in the size and intensity of brown eggshell spots could not be shown in blue tits [23], and an increase in spot intensity even occurred in great tits [24], which is at odds with the structural function hypothesis. With regards to the SSECH for brown protoporphyrin-based pigmentation, there are two contrasting predictions that pertain to the pro-oxidant properties of protoporphyrins, i.e. inductors of oxidative stress [25]. First, a large amount of protoporphyrins in eggshells (i.e. causing more spots, bigger spots and/or browner spots), could indicate high female and egg quality if it reflects the female's high anti-oxidant capacity (or oxidative tolerance) to withstand the pro-oxidants or to transfer them efficiently to the eggshell [1]. Second, and conversely, a large amount of protoporphyrins in eggshells could indicate poor female health status and egg quality if the female suffers from the physiological stress induced by a high level of circulating protoporphyrins [7]. The first prediction, namely that browner eggs should reflect high-quality females, has received mixed support (reviewed in Table S1). Moreover despite their opposite reasoning, both predictions have received some support in a single species, the blue tit [7], [26]. However, the two studies are difficult to compare as they used different methods to measure eggshell spottiness (Table S1).

Next to experimental approaches (e.g. [27]), the best way to understand the function(s) of brown eggshell pigmentation is by implementing comparable and accurate measurements across studies. This is a larger scale problem ranging beyond blue tits as it extends to all species where brown pigmentation has been studied with different methods to date (e.g. [22], [23], [28], see also Table S1).

The first aim of our study was thus to explore the correlations between the following measurements of eggshell coloration (Table S1): spectrometric measurements directly on the eggs, the percentage of eggshell surface covered with spots and digital color values (e.g. [29]), both extracted from digitized pictures, and the pigmentation indices of Gosler *et al.* [22], [28]. Then, to test if a browner eggshell indicates a high or low maternal investment in the egg, we measured its relationship with the transfer of maternal antibodies and/or carotenoids to the egg yolk. In addition to eggshell spottiness, several indicator traits of parental quality are known to potentially affect the maternal transfer of antibodies and carotenoids. In our analyses, we thus included a female's age, condition and plumage coloration as well as her partner's plumage coloration as these factors have been shown to be linked to maternal investment in reproduction in blue tits [30], [31].

## Materials and Methods

### 1. Ethics Statement

All procedures followed the French laws. We conducted the work under permits given by the “Préfecture” of Hérault and the Regional Direction of Environment DIREN committee to our research program (permit 2006-01-2014), to our CEFE institute (permit B34-172-11) and to ourselves (permit 3467).

### 2. Breeding and morphometric variables

The study population of blue tits is located in southern France in the “La Rouvière” wood (43°40′N, 03°40′E), where breeding parameters (onset of egg laying, laying order, and clutch size) are collected yearly through the routine inspection of nest boxes [32].

In 2007, we removed the whole clutch of 42 breeding pairs (same sampling as in [33]) just after clutch completion (mean = 1.9 days after the start of incubation period, range = 0–6). We captured parents (41 females and 29 males) on the same day (except for two pairs) at nest boxes between 6 and 20 days after the first egg was laid (mean = 11 days for both females and males). Bird tarsus length was measured to the nearest 0.01 mm. Breeders' age (yearling vs. adult) was determined through the color of the wing coverts.

### 3. Color measurements and variables

#### Eggshell coloration by spectrometry

Our spectral measures specifically distinguished between spot patterns (mainly protoporphyrins) and eggshell ground color (a mixture of compounds with different physiological, physical, or spectral properties) as both colors are likely to be determined by different mechanisms [1], [3]. Thus to avoid mixing brown with white background, we only measured spots larger than the spectrometer probe (Ø = 2.2 mm). In addition to spectral measures of brown spots, we used measures of white eggshell coloration as a covariate in the predictive models of yolk compounds (see below). Indeed, its high between-clutch variability in our study population [33] suggests it may allow clutch discrimination and may reflect female or egg quality. Spectral measures have been described in details in [33]. Briefly, we measured the white coloration of all eggs (479 from 42 clutches) and the brown spots on a subsample of 31 eggs from 30 clutches with an Ocean Optics USB4000 with xenon lamp PX2 spectrometer (range: 300–700 nm) and 200-microm fibre optic probe. All measurements were made perpendicular to the eggshell surface using the probe mount with a back rubber cap to exclude ambient light. The probe was held at a fixed distance of 2 mm from the eggshell surface. We generated reflectance data relative to a white (WS1 ocean optics) and dark (black felt background) standard. For each egg and color variable, we computed the mean of five reflectance spectra (figure S1 in [33]).

Color spectra information was extracted using Avicol software v4 [34]. From the color spectra, we calculated three color parameters for the brown and white color parts of the eggshell separately: brightness, UV chroma, and chroma. Both brightness and UV chroma are likely important for visual detection in nest cavity [33]. They both correspond well with the shape of reflectance spectra [35] and were computed accordingly. For both brown and white color parts, brightness was the mean reflectance over the range 300–700 nm (computed as the area under the curve divided by the width of the interval 300–700 nm to include the whole range of bird sensitivity) and UV chroma was computed as (R_300_–R_400_)/(R_300_–R_700_). We computed brown spot chroma as (R_539–700_)/(R_300–700_) since protoporphyrins, the pigments responsible for brown pigmentation, have three main absorption peaks at 539, 589, and 643 nm [36], and white chroma as (R_700_-R_300_)/(R average _300–700_). The repeatability [37] of these measurements taken at different points of the eggshell was significant (white: 0.41>*R*>0.81, all *F*_478,1898_>4.5, *P*<0.001; brown: 0.19>*R*>0.47, all *F*_30,123_>2.2, *P*<0.01).

All computed brown spectral variables were inter-correlated (brown spot brightness and UV chroma: Pearson *r*_29_ = 0.46, *P*<0.01, brown spot brightness and chroma: *r*_29_ = −0.59, brown spot chroma and UV chroma: *r*_29_ = −0.91, both *P*<0.001). Given the very high correlation between brown spot chroma and UV chroma, we only kept the former for further analyses as it is likely to best reflect protoporphyrin content. Therefore we only kept spectral chroma and brightness of brown spots in our analyses. In contrast, since the white spectral variables of brightness and UV chroma were not correlated with each other (Spearman *rho*_477_ = 0.03, *P* = 0.5), and were both significantly but weakly correlated with white chroma (*rho*_477_ = −0.43, *P*<0.001, and 0.14, *P*<0.01, respectively), we therefore kept the three white spectral parameters in our analyses. Brown spectral variables did not correlate with white spectral ones (−0.30<all *r*_29_<0.23, all *P*>0.1).

#### Brown-spotted eggshell surface from digitized pictures

Indoors, we took standard photographs of 474 eggs (5 got broken): each egg was photographed twice on the same side while placed on a black tissue background with a ruler (screen precision 1 pixel), with a digital camera (Nikon D70S) equipped with a macro lens (AF-S VR105 F/2.8) and a ring flash (macro Nikon SB-29s; manually set to 1/32). Using ImageJ software v1.38× [38], we extrapolated the total eggshell surface and the spotted surface from pictures of one eggshell half. With the implemented plug-in ‘Threshold color’, the color parameters (i.e. brightness, saturation, hue) were adjusted for each egg until the separation of the spotted surface from the white matrix was achieved. The area of the spotted surface was then measured. The same procedure was used to calculate the total surface of the egg. It allowed estimating the percentage of eggshell surface covered with brown spots ( = spotted surface/eggshell surface), named ‘brown-spotted surface’ thereafter, and the egg volume ( = 0.507×Length×Breadth^2^ [39]). The latter was used as a covariate in the predictive models of yolk compounds (see below) as absolute amounts of yolk antibodies and carotenoids can covary with egg size [10]. Our method to extract the spotted surface was reliable as measurements were significantly repeatable for 266 eggs (*R* = 0.95, *F*_265,266_ = 35.6, *P*<0.001) for which we used the mean of both pictures. Thus for the remaining eggs, we used only one of the two pictures.

#### Eggshell coloration from digitized pictures

We calculated the digital hue, saturation, and brightness of brown spots and white eggshell coloration derived from RGB (Red, Green, Blue) and HSB (Hue, Saturation, Brightness) color spaces. H describes the dominant wavelength of the color, S is the amount of H mixed into the color, and B the amount of light in the color [40]. RGB and HSB color spaces have recently been criticized (see extensive guidelines in [41], [42]), but here we present these color scores for comparative purposes with prior work (Table S1). We extracted RGB values of 10 randomly selected spots (mean ±1 SD = 707±996 square pixels, *n* = 7400 measures) and 5 randomly selected white eggshell areas (1762±1331 square pixels, *n* = 3700) per picture (*n* = 474 first pictures+266 second pictures) with ‘Wand Tool’ (mode: 8-connected, tolerance: 8) in ImageJ software. The eggs were all photographed on the same black background that we used as a reference to standardized color measurements between pictures. The black reference consisted of a rectangle of equal size (601600 square pixels) and position (from top left to right) among all pictures. We obtained HSB values from RGB ones in R v. 2.15.1 with the rgb2hsv function [43]. For the brown and white color parts of the eggshell separately, we then standardized H, S, and B of each picture based on the deviance of H, S, and B mean (calculated from the full sample), respectively. Following [44], the standardized value of H of each spot from one picture was equal to “H of each spot of the focal picture” minus the deviation, which was the absolute value of |“H mean of the black reference of all pictures” minus “H of the black reference of the focal picture”|. Identical calculations were done for S and B of the brown color part and H, S, and B of the white color part. We used the mean of the repeated measurements per color part and per egg as they were significantly repeatable (brown: 0.53>*R*>0.99, all *F*_473,4266_>12.1; white: 0.89>*R*>0.96, all *F*_473,1896_>44.8; all *P*<0.001). Our method to measure HSB was reliable as shown by the significant repeatability between both pictures of 266 eggs for which we used the mean (brown: 0.39>*R*>0.74; white: 0.36>*R*>0.69; all *F*_265,266_>2.1, *P*<0.001). Brown digital variables were inter-correlated (H-S, H-B, and S-B: *rho*_472_ = −0.38, 0.52, and −0.57, respectively; all *P*<0.001), as were white digital ones (H-S, H-B, and S-B: *rho*_472_ = −0.50, 0.62, and −0.35, respectively; all *P*<0.001). Brown digital variables did correlate with white digital ones (−0.49<all *rho*_472_<0.95, all *P*>0.001). Given the very high correlations of digital H and B between brown spots and white eggshell coloration (rho_472_ = 0.76 and 0.95, respectively), we only kept the three brown digital variables and the digital S of white eggshell coloration in our analyses.

#### Brown eggshell pigmentation indices from digitized pictures

**Figure 1:**
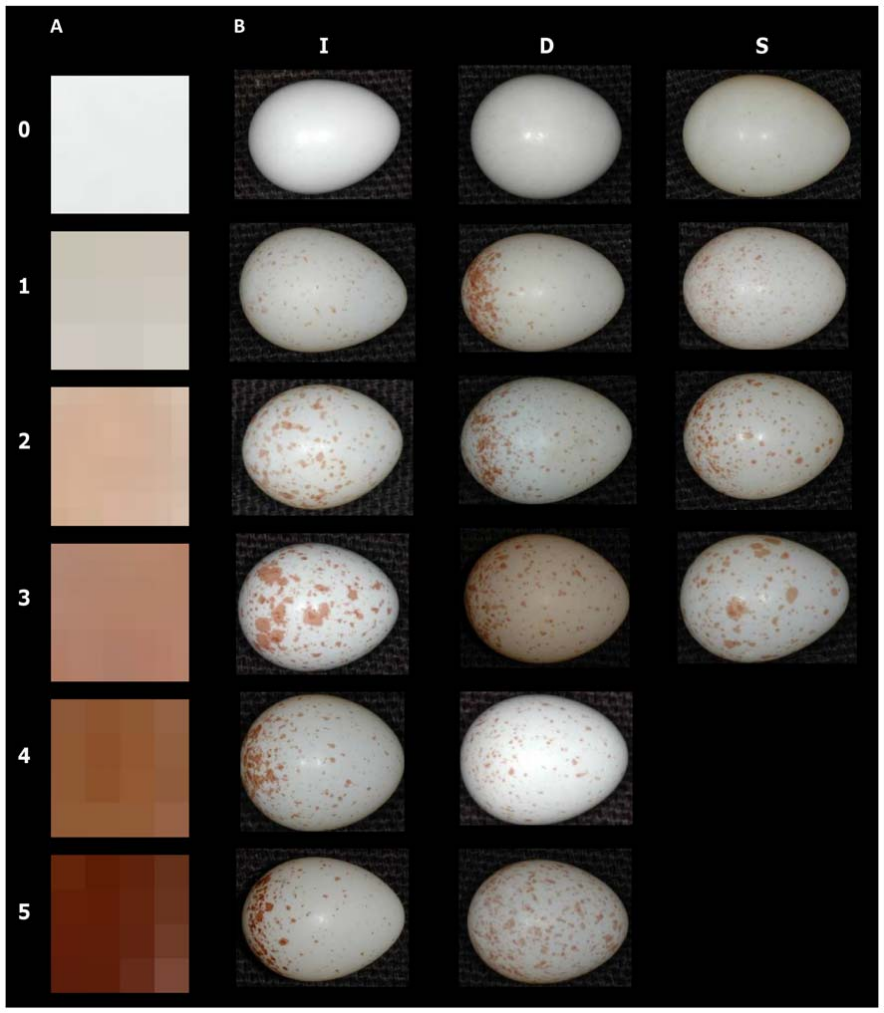
Between-female variation of eggshell spottiness in the studied blue tit population in southern France. (A) Intensity code, (B) standard of comparison. Intensity I: 0, no spot, 1, feint, 2, pale, 3, medium, 4, some intense spots, 5, intense. Distribution D (approximate percentage spotting in one-half, typically the blunt end): 0, no spot, 1, >81%, 2, 71–80%, 3, 61–70%, 4, 51–60%, 5, 50%. Spot size S: 0, no spot, 1, small, 2, medium, 3, large. Rows represent increasing values from top to bottom. After [26], [28]. Egg pictures by AG.

Following Gosler *et al.* [22], [28], three observers (MJH, PP, AG) scored the brown eggshell pigmentation pattern based on brown spot intensity (scored 0 to 5), distribution (0 to 5), and size (0 to 3) from the first picture taken per egg (Figure 1); scoring was blind with respect to clutch identity and laying order. Intermediate values (e.g. 1.5, 2.5) were also interpolated, giving 11 classes for intensity and distribution, and 7 for size. For scoring, an intensity code (spot enlargement printed on photo paper) was available in addition to the standard of comparison (Figure 1). We computed the mean of the three scores per egg as observer scores were highly correlated (intensity: 0.59>Spearman *rho*_472_>0.75, distribution: 0.85>*rho*_472_>0.88, size: 0.74>*rho*_472_>0.83, all *P*<0.001).

The three indices were inter-correlated (intensity-distribution, intensity-size, and distribution-size: *rho*_472_ = −0.61, 0.47, and −0.41, respectively; all *P*<0.001). For comparison with previous work (Table S1), we calculated the principal components PC1 (explaining 65.6% of the total variance, with intensity = 0.615, distribution = −0.584, size = 0.530) and PC2 (explaining 21.3% of the total variance, with intensity = −0.203, distribution = 0.531, size = 0.822) from the correlation matrix. As our PCs showed similar loadings to the ones in [22], [28], they were labeled accordingly, namely brown pigment darkness and spread respectively. Increasing pigment darkness (PC1) represented increasing spot intensity and size, and less evenly distributed spots over the eggshell surface. Increasing pigment spread (PC2) represented increasing spot size and more evenly distributed spots over the eggshell surface.

#### Plumage coloration by spectrometry

We calculated brightness and hue of the UV-blue cap, and brightness and chroma of the yellow collar (see Methods S1 and [30]).

### 4. Egg yolk compounds

Among the 479 eggs from 42 clutches, we analyzed the yolk content of 72 eggs from 35 clutches. We analyzed two eggs per clutch (apart from one clutch where four eggs were used). This level of sampling is sufficient to obtain a good estimate of female investment in the whole clutch as shown by the high within-clutch repeatabilities of yolk compounds reported in bird species (e.g. for antibodies in kittiwakes *Rissa tridactyla* [45], carotenoids in blue tits [46]). One of the two eggs was the second one in the laying sequence (mean laying rank ±1 SD = 1.7±0.6, range = 1–4, *n* = 36) and the other the fourth (4.1±0.9, range = 2.5–6, *n* = 33). For analyses, we used the exact laying order. When egg rank could not be unequivocally determined (eggs found on the same day), they received an intermediate rank (eggs were numbered with a drawing pencil). Yolks were separated from the albumen, homogenized, and stored at −20°C until analyses.

#### Immunological assays

To extract immunoglobulins, the yolks were weighed and an equal weight of water added. The samples were kept overnight at +4°C, and then centrifuged at 16000 g for 45 min, at +4°C. The supernatants were collected and immunoglobulin concentrations in the eggs extracts were determined with the ELISA method. Briefly, 96-well microplates (Immuno Plate Maxisorp, Nunc Co., Nunc A/S, Roskilde, Denmark) were first coated overnight at +4°C with commercial antichicken IgG antibody (C-6409, Sigma Chemical Co., St. Louis, MO, USA). The wells were then saturated for 1 hour with 1% bovine serum albumin (BSA, Roche Diagnostics GmbH, Manheim, Germany). After washing, samples diluted at 1∶1000 with 1% BSA/PBS were incubated for 3 hours at room temperature. After washing, alkaline phosphatase conjugated antichicken IgG antibody (A-9171, Sigma Chemical Co.) was added and the plates were incubated overnight at +4°C (dilution of 1∶2000). Finally p-nitrophenyl phosphate (Sigma 104 Phosphatase Substrate) was added. After 45 min, the optical density was read at 405 nm with a spectrometer (Multiskan Ascent, Therma Oy, Finland). Serial dilution of the positive control was performed in order to generate the standard curves. Immunoglobulin levels are expressed as the optic density (OD) of the resulting solution, which is a relative measure of antibody concentration in the plasma samples. Each sample was run in duplicate, on two different plates. The repeatability of measurements from the two different ELISA-plates was high (*R* = 0.81, *F*_71,72_ = 9.7, *P*<0.001) and we thus used the mean.

#### Carotenoid measurements

Between 50 and 100 mg of egg yolk were weighed and mixed with 0.79 times their weight of acetone (1 ml of acetone for 100 mg of yolk). Samples were kept overnight at −20°C, and then centrifuged at 13000 g during 5 min at +4°C. The OD of 125 µl of the supernatant was determined at 450 nm. We used serial dilution of a commercial solution of carotenoids (Sigma X-6250) to generate the standard curve and determine the relationship between OD values and carotenoid concentrations expressed in µg per gram of egg yolk. Samples were run in duplicate and we took the mean of the two highly repeatable measures (*R* = 0.99, *F*_70,71_ = 262.5, *P*<0.001). One extreme outlier (185.2 µg.g^−1^ versus a range of 8.2 to 74.7 µg.g^−1^ for the 71 other eggs) was not taken into account in any of the analyses below.

#### Yolk-compound relationships

Yolk antibody and carotenoid concentrations were neither correlated at the level of the clutch (Pearson *r*_33_ = 0.08, *P* = 0.7) nor at the level of the individual egg (Spearman *rho*_69_ = 0.09, *P* = 0.5). At the egg level, when nest identity was used as a random factor, the linear mixed-model residuals of each compound were also not correlated over the laying sequence (Pearson *r*_66_ = −0.03, *P* = 0.8). The latter relationship was examined to account for the fact that egg compounds can vary with laying order (e.g. [47], [48], [49]). Yolk antibodies and carotenoids were therefore treated as independent variables in this study.

### 5. Statistical analyses

First, we checked the relationships among brown eggshell-color variables. We paid particular attention to the correlations between the pigmentation indices - used to test both the potential structural and signaling functions of spottiness (e.g. [22], [28], Table S1) - and the quantitative measurements of brown coloration by spectrometry (i.e. spectral chroma and brightness of brown spots) and with computer-analyzed pictures (digital hue, saturation, and brightness of brown spots, and brown-spotted surface) - used to assess the potential signaling function (Table S1).

Second, we investigated which parameters (breeding parameters, egg, female, or male traits) best predicted yolk antibody and carotenoid concentrations, which were square root- and log-transformed respectively to achieve normality (Shapiro-Wilk tests on full model residuals: all *W*>0.95, *P*>0.05). For this, we ran two-tailed (*α* = 0.05) linear-mixed models LMMs (Type III) fitted by maximizing the log-likelihood in R v. 2.15.1 under the nlme package [43] and with nest identity as a random factor. The ideal analysis to determine the best predictors of yolk antibody and carotenoid concentrations should include all parameters in a single model to evaluate the degree of influence of each of them. However, because the male sample was smaller than the female one as well as to avoid a decrease in the degrees of freedom and model over-parametization (and Type I errors [50]), we built two types of statistical models: (i) an egg- and female-trait model testing the female traits that may best reflect egg content to males and (ii) a male-trait model testing for differential allocation by females in response to variation in male quality. We also present the full models in Tables S2, S3.

The fixed factors common to both model types were egg laying order, egg volume, laying date (day 1 = 01/03/2007), clutch size residuals derived from regression of clutch size on laying date (as both parameters were significantly correlated: *rho*_32_ = −0.37, *P* = 0.03), and the number of days during which the clutch was incubated (as yolk carotenoids may decrease when the developing embryo starts mobilizing the resource [17]).

The fixed factors specific to (i) were female traits - tarsus length, age (yearling or adult), blue feather brightness and hue, and yellow feather brightness and chroma - and eggshell-color traits – the two first principal components of brown pigmentation pattern, i.e. darkness and spread respectively, the brown-spotted surface, the digital hue, saturation, and brightness of brown spots, the digital saturation of the white eggshell, and the spectral brightness, UV chroma, and chroma of the white eggshell. These brown and white eggshell-color variables did not strongly correlate with each other (−0.40<all *rho*_472_<0.47, all *P* from 0.7 to <0.001), and the correlations among the spectral and digital white eggshell-color variables were weak (−0.17<all *rho*_472_<0.14, all *P* from 0.8 to <0.001). The fixed factors specific to (ii) were male traits (same as female ones).

We used a stepwise backward selection procedure on fixed factors and kept the model with the best fit based on AIC and the log-likelihood ratio test. As we could not capture all birds (11 males and 1 female out of 35 couples were not captured) nor ascertain all laying orders (3 eggs out of 72), sample sizes sometimes differ between analyses.

In the above predictive models, we could not use brown spectral variables as fixed factors since the measured eggs were often different from the ones for which we measured yolk content (only two eggs were measured for both variable types). Nevertheless, using another sample collected in 2008, we found significant within-clutch repeatabilities for the brown spectral variables (spectral chroma of brown spots: *F*_9,90_ = 3.8, *R* ±1 SE = 0.22±0.11; spectral brightness of brown spots: *F*_9,90_ = 6.8, *R* ±1 SE = 0.37±0.13; both *P*<0.001, *n* = 100 eggs from 10 clutches). Thus in our 2007 sample, for each clutch with at least one egg measured for brown spectrum and one for yolk content, we gave the value of the egg measured for brown spectrum (or the mean when two eggs were measured) to the two eggs measured for yolk content (48 eggs from 23 clutches). Given the substantial decrease in sample size, we tested the link of the brown spectral variables to each yolk compound by adding them as factors in the previously obtained minimal models of higher sample sizes.

We report within-clutch repeatability estimates of egg traits following [37] and their standard error following [51]. All estimates were calculated from the yolk compound sample (*n* = 72 eggs from 35 clutches) for a straightforward comparison between the internal and external egg measurements.

## Results

### 1. Relationships among brown eggshell-color traits

**Figure 2:**
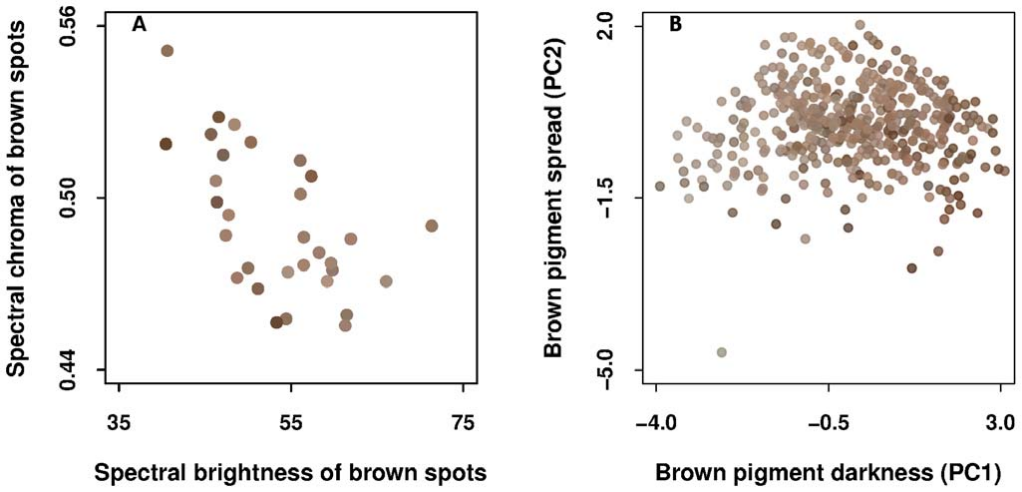
Relationships of spot RGB scores (i.e. dots' color) with their spectral measures and pigmentation indices. (A) The two brown spectral variables, (B) the two principal components obtained from the three brown eggshell pigmentation indices. We increased the transparency of points (alpha = 200) to improve the visibility of the 474 eggs from 42 clutches in (B). We measured the spectral coloration of the spots on a subsample of 31 eggs from 30 clutches in (A). The brown spots become browner (in response to digital saturation and hue) and darker (digital brightness) as spectral brightness decreases (A) and PC1 values increase (B). There are not such relationships with spectral chroma (A) and PC2 values (B).

The spectral and digital measures of brown spots did not entirely reflect each other as their correlations were all below 50% (Table 1). All digital measures significantly correlated with the spectral brightness but not with the spectral chroma of brown spots. Figure 2A shows how the spots become browner (saturation and hue components) and darker (brightness component) as their spectral brightness decreases. Figure 2A also shows the absence of such clear relationships with spectral chroma of brown spots.

**Table 1:** Correlations between brown eggshell-color traits.

Trait 1	Trait 2	Pearson *r* or Spearman *rho*
Spectral brightness of brown spots	Brown-spotted surface	−0.30^a^^, ^ b
	Digital hue of brown spots	0.38* a ^, ^ b
	Digital saturation of brown spots	−0.48** b
	Digital brightness of brown spots	0.41* b
	*Brown pigment darkness (PC1)*	−0.39* b
	*Brown pigment spread (PC2)*	−0.23^b^
	*Brown spot intensity*	−0.45* b
	*Brown spot distribution*	0.10^b^
	*Brown spot size*	−0.43* b
Spectral chroma of brown spots	Brown-spotted surface	0.24^a^^, ^ b
	Digital hue of brown spots	−0.16^a^^, ^ b
	Digital saturation of brown spots	0.34°^b^
	Digital brightness of brown spots	−0.26^b^
	*Brown pigment darkness (PC1)*	0.32^b^
	*Brown pigment spread (PC2)*	0.36* b
	*Brown spot intensity*	0.37* b
	*Brown spot distribution*	−0.004^b^
	*Brown spot size*	0.46* b
Brown-spotted surface	Digital hue of brown spots	0.02^a^^, ^ c
	Digital saturation of brown spots	0.27*** a ^, ^ c
	Digital brightness of brown spots	−0.14** a ^, ^ c
	*Brown pigment darkness (PC1)*	0.55*** a ^, ^ c
	*Brown pigment spread (PC2)*	0.48*** a ^, ^ c
	*Brown spot intensity*	0.37*** a ^, ^ c
	*Brown spot distribution*	−0.31*** a ^, ^ c
	*Brown spot size*	0.76*** a ^, ^ c
Digital hue of brown spots	*Brown pigment darkness (PC1)*	−0.19*** a ^, ^ c
	*Brown pigment spread (PC2)*	0.25*** a ^, ^ c
	*Brown spot intensity*	−0.28*** a ^, ^ c
	*Brown spot distribution*	0.17*** a ^, ^ c
	*Brown spot size*	0.05^a^^, ^ c
Digital saturation of brown spots	*Brown pigment darkness (PC1)*	0.66*** a ^, ^ c
	*Brown pigment spread (PC2)*	−0.12* a ^, ^ c
	*Brown spot intensity*	0.79*** a ^, ^ c
	*Brown spot distribution*	−0.44*** a ^, ^ c
	*Brown spot size*	0.34*** a ^, ^ c
Digital brightness of brown spots	*Brown pigment darkness (PC1)*	−0.39*** a ^, ^ c
	*Brown pigment spread (PC2)*	0.23*** a ^, ^ c
	*Brown spot intensity*	−0.51*** a ^, ^ c
	*Brown spot distribution*	0.29*** a ^, ^ c
	*Brown spot size*	−0.09°^a^^, ^ c

Significant values are in bold. The traits assessed by human eyes are in italics. *P*-value<0.001***, <0.01**, <0.05*, <0.07°.

a Spearman *rho*.

b 31 eggs from 30 clutches as the spectral coloration of the spots was measured on a subsample.

c 474 eggs from 42 clutches.

Although neither spectral brightness nor chroma of brown spots entirely reflected the brown eggshell-color variables as assessed by human eyes (all correlations <50%), both brown spectral variables explained a significant part of the variation in both brown spot intensity and size (Table 1). Consequently, spectral brightness of brown spots significantly correlated with brown pigment darkness (PC1) (Pearson *r*_29_ = −0.39, *P* = 0.029), with most of the variation attributable to brown spot intensity, and spectral chroma of brown spots was significantly correlated with brown pigment spread (PC2) (*r*_29_ = 0.36, *P* = 0.047; Table 1), with most of the variation being due to brown spot size (Table 1).

The brown-spotted surface as digitally extrapolated significantly and strongly correlated with brown spot size (76%) and to a lesser, but significant, extent (<40%) with brown spot intensity and distribution (Table 1). Consequently, it significantly correlated with both principal components, i.e. brown pigment darkness (PC1) and spread (PC2) (Spearman *rho*_472_ = 0.55 and 0.48, respectively, both *P*<0.001).

Digital hue of brown spots significantly, but weakly, correlated with brown spot intensity and distribution (<30%), and accordingly to both principal components (<30%), but not at all with spot size and surface (Table 1). Digital saturation and brightness of brown spots significantly and strongly correlated with brown spot intensity (79 and 51%, respectively) and to a lesser, but significant, extent (<50%) with brown spot distribution and size (Table 1). Consequently, digital saturation and brightness of brown spots correlated more strongly with brown pigment darkness (PC1) (*rho*_472_ = 0.66 and −0.39, respectively, both *P*<0.001) than with brown pigment spread (PC2) (*rho*_472_ = −0.12 and 0.23, respectively) or brown-spotted surface (*rho*_472_ = 0.27 and −0.14, respectively). Accordingly, Figure 2B shows how the spots become browner (saturation and hue components) and darker (brightness component) as PC1 values increase, and the absence of such clear relationships with PC2 values.

For the 72 eggs sampled for yolk compounds, most estimators of eggshell coloration as well as egg volume were significantly repeatable within clutches (0.27<*R*<0.77, 1.7<*F*_34,37_<7.8, all *P*<0.05). Within these samples, digital hue of brown spots and digital saturation of the white eggshell were not repeatable within clutches (−0.05<*R*<0.17, 0.9<*F*_34,37_<1.4, both *P*>0.15), but they were repeatable when taking the full egg sample into account (0.24<*R*<0.35, 4.6<*F*_41,432_<7.1, both *P*<0.001).

### 2. The predictors of yolk compounds

Untransformed yolk concentrations were 1.43±0.31 OD (mean ±1 SD) for antibodies (range = 0.74–2.19, *n* = 72 eggs) and 30.1±14.3 µg.g^−1^ for carotenoids (range = 8.2–74.7, *n* = 71 eggs). Yolk antibody and carotenoid concentrations were both highly repeatable within clutches (*F*_34,37_ = 10.3, *R* ±1 SE = 0.82±0.05 and *F*_34,36_ = 4.1, *R* ±1 SE = 0.60±0.11, respectively; both *P*<0.001).

**Figure 3:**
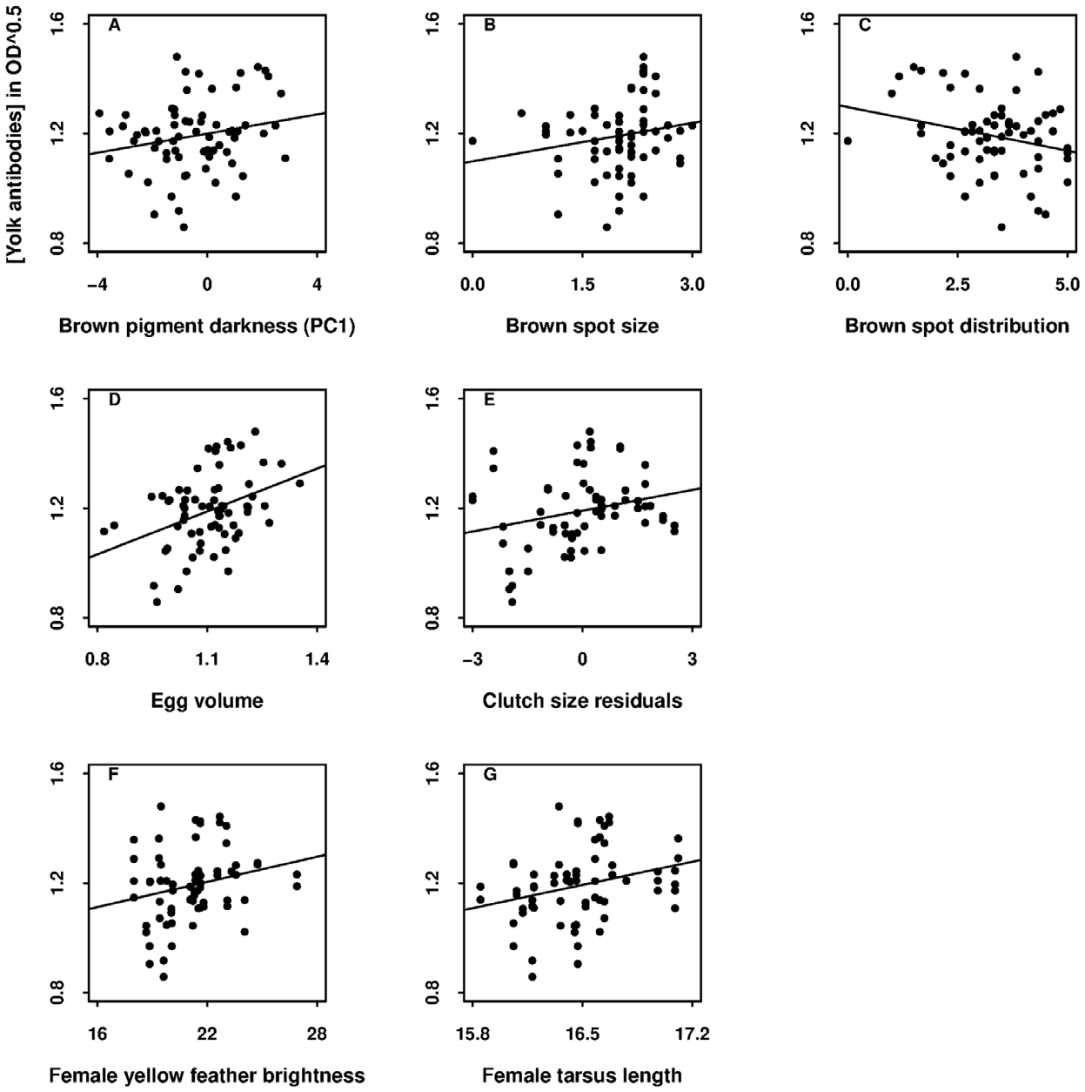
The significant predictors of yolk antibody concentration in OD^0.5^ in blue tit eggs. They were (A) brown pigment darkness (PC1), (B) brown spot size and (C) distribution of eggshells, (D) egg volume, (E) residuals of clutch size on laying date, (F) yellow feather brightness, and (G) tarsus length of females. Plots show the data used in the egg- and female-trait model (Table 2).

Different parameters predicted antibody and carotenoid concentrations in egg yolks. In the model (i) based on the higher sample size with egg and female traits as predictors, yolk antibody concentration significantly increased with eggshell brown pigment darkness (PC1), egg volume, and clutch size residuals (Tables 2, S2; Figure 3A,D–E; see Results S1). The first relationship means that the eggs with more intense and larger spots concentrated at their broad ends had higher antibody concentration. In addition, two female traits significantly and positively predicted antibody concentration, namely yellow feather brightness and tarsus length (Tables 2, S2; Figure 3F–G). In contrast, in the model (ii), none of the male traits significantly predicted yolk antibody concentration (Tables 2, S2). In this male-trait model, yolk antibody concentration was still significantly related to egg volume and clutch size residuals.

**Table 2:** Yolk antibody concentration of blue tit eggs in relation to egg, female, and male traits.

Model terms	Estimate ±1 SE	*F*	d.f.	*P*
**Egg and female traits** 1				
Egg volume	0.375±0.128	8.6	1,32	0.006
Yellow feather brightness	0.023±0.009	7.0	1,29,32	0.013
Pigment darkness (PC1)	0.017±0.007	6.0	1,32	0.020
Residuals of clutch size on laying date	0.029±0.013	5.2	1,29,32	0.030
Tarsus length^3^	0.107±0.054	3.9	1,29,32	0.058
**Male traits** 2				
Egg volume	0.411±0.137	9.1	1,22	0.006
Residuals of clutch size on laying date^3^	0.044±0.022	4.2	1,21,22	0.054

Separate mixed model analyses were performed for egg and female traits and male traits.

1 67 eggs from 33 clutches.

2 46 eggs from 23 clutches.

3 Parameters that could not be removed from the final model based on significant log-likelihood ratio tests (both *P*<0.05).

The loadings of the three pigmentation indices were almost equal in PC1 (see methods), making difficult to know which index or indices may best predict yolk antibody concentration. In three separate models in which we excluded the two PCs but included one of each of the three indices, we found that brown spot size (Estimate ±1 SE = 0.041±0.019, *F*_1,32_ = 4.5, *P* = 0.04) and distribution (−0.025±0.010, *F*_1,32_ = 6.0, *P* = 0.02), but not intensity (0.015±0.012, *F*_1,32_ = 1.6, *P* = 0.2), significantly predicted yolk antibody concentration. The previously found predictors remained identical, i.e. effects of clutch size residuals, egg volume, yellow feather brightness, and tarsus length of females (statistics not shown). Eggs with large spots concentrated at their broad ends had a higher concentration of antibodies, while eggs with smaller, more evenly distributed spots had lower antibody concentrations (Figure 3B–C).

**Figure 4:**
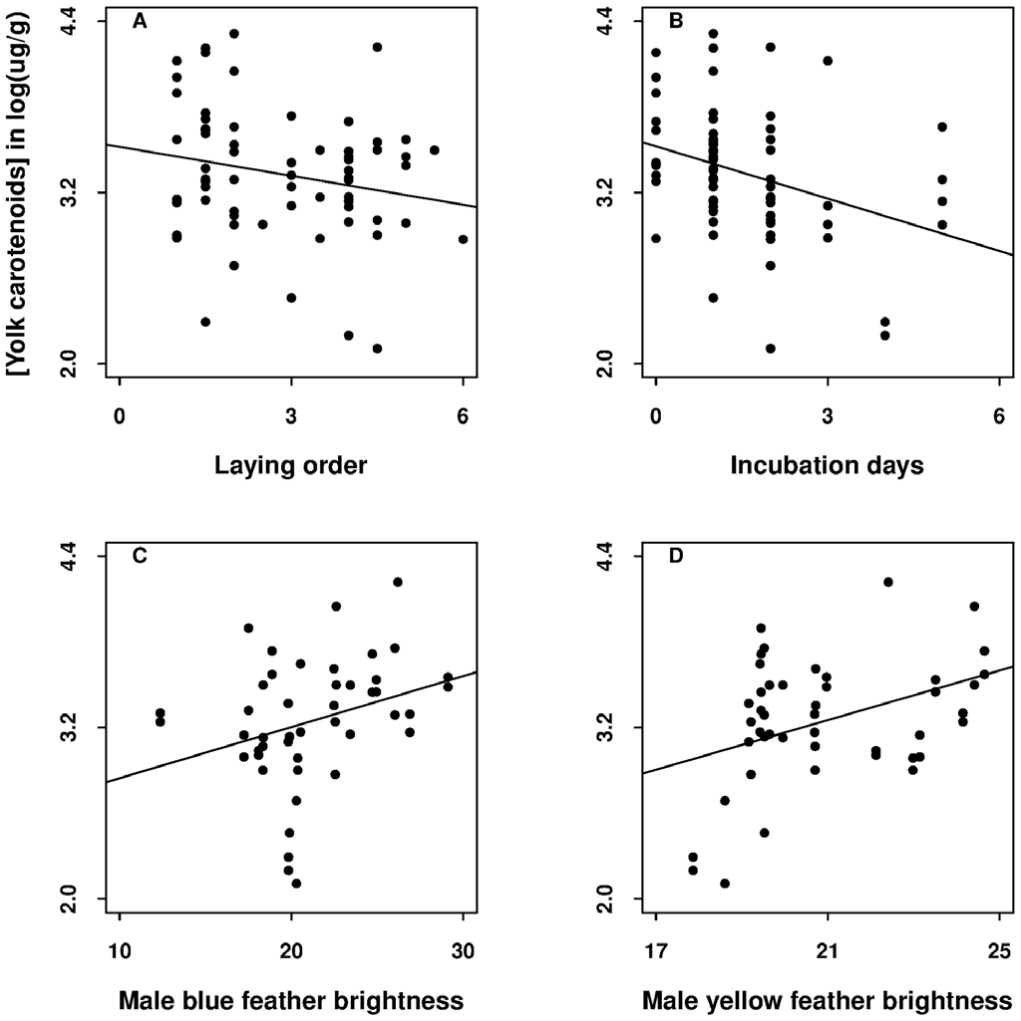
The significant predictors of yolk carotenoid concentration in log(µg.g^−1^) in blue tit eggs. They were (A) laying order, (B) number of days of clutch incubation, (C) blue feather brightness, and (D) yellow feather brightness of males. Plots a–b show the data used in the egg- and female-trait model, c-d in the male-trait model (Table 3).

Yolk carotenoid concentration significantly decreased with laying order and the length of clutch incubation in both analyses (i) and (ii), i.e. with egg and female traits and with male traits as the only predictors (Tables 3, S3; Figure 4A–B). Two male traits positively and significantly predicted yolk carotenoid concentration: blue and yellow feather brightness (Tables 3, S3; Figure 4C–D). None of the eggshell-color or female traits significantly predicted yolk carotenoid concentration (Tables 3, S3). The egg- and female-trait models using the pigmentation indices instead of the two PCs as predictors led to the same final model (statistics not shown).

**Table 3:** Yolk carotenoid concentration of blue tit eggs in relation to egg, female, and male traits.

Model terms	Estimate ±1 SE	*F*	d.f.	*P*
**Egg and female traits** 1				
Laying order	−0.084±0.025	11.4	1,32	0.002
Days of clutch incubation	−0.148±0.060	6.0	1,31,32	0.020
**Male traits** 2				
Blue feather brightness	0.051±0.017	9.4	1,19,21	0.006
Yellow feather brightness	0.089±0.032	7.7	1,19,21	0.012
Days of clutch incubation	−0.210±0.081	6.7	1,19,21	0.018
Laying order	−0.054±0.026	4.4	1,21	0.049

Separate mixed model analyses were performed for egg and female traits and male traits.

1 66 eggs from 33 clutches.

2 45 eggs from 23 clutches.

Neither antibody nor carotenoid concentrations were significantly predicted by brown spots' spectral chroma (antibodies: *F*_1,16,21_ = 0.3, *P* = 0.6; carotenoids: *F*_1,18,21_ = 0.6, *P* = 0.4) or brightness (antibodies: *F*_1,17,21_ = 0.6, *P* = 0.4; carotenoids: *F*_1,19,21_ = 0.9, *P* = 0.3) when these spectral variables were added to the minimal models (the statistics given here were based on egg- and female-trait models in Tables 2–3).

## Discussion

This study aimed at testing if several characteristics of eggshell spottiness frequently used in the literature reflected similar or different information on the amount of two maternally-derived yolk compounds, antibodies and carotenoids, transferred to blue tit eggs. First, we tested the relationships between these target eggshell-color characteristics and found some partial correlations between the spectral and digital (HSB color space) measures of brown eggshell coloration, the spotted surface, and pigmentation indices, highlighting the necessity of implementing consistent and comparable quantitative measurements in future studies. We then measured the correlations between several eggshell-color variables and the level of yolk antibodies and carotenoids in multivariate regressions including several parental traits and breeding parameters that could potentially affect maternal transfer of compounds to the eggs. We found that yolk antibody concentration increased with increasing brown pigment darkness (PC1) (a composite measure of three pigmentation indices that is heritable on the female line in great tits [28]), and in particular with the pigmentation indices measuring the size and the distribution of brown spots over the egg surface. In contrast, eggshell coloration had no relationship with yolk carotenoid content. Lastly, we found that levels of both egg yolk compounds were correlated with either female or male plumage coloration: females laying eggs with more yolk antibodies had brighter yellow feathers and females laying eggs with more yolk carotenoids were mated to males with brighter blue and yellow feathers.

The correlations we found between the different measures of brown eggshell coloration validates, to some extent, the utilization of the easy-to-use, quick, and inexpensive pigmentation indices first proposed by Gosler *et al.* [22], [28] in the context of the structural function hypothesis. Both brown pigment darkness (PC1) and spread (PC2) showed positive 50% correlations with brown-spotted surface. PC1 also showed a negative 40% correlation with spectral and digital brightness of brown spots and a positive 66% correlation with digital saturation of brown spots, and PC2 a positive 40% correlation with spectral chroma of brown spots. However, quantitative measurements are easier to compare among studies and to manipulate experimentally than the integrative principal components PCs derived from Gosler's pigmentation indices as PCs' loadings can differ between studies (e.g. [22], [26], this study). Moreover, PC1 almost equally reflected brown spot intensity, size, and distribution, but among these three pigmentation indices only spot size and distribution predicted yolk antibody concentration. Taken together, this leads us to recommend the following parameters when assessing both signaling and structural functions of eggshell spottiness, namely (i) spectral chroma of brown spots (specifically set-up to reflect protoporphyrin content), (ii) brown spot size (a Gosler's index that predicted yolk antibody concentration) or its strong correlate, the spotted surface (which correlated with both PCs and is probably less dependent on human subjectivity, see also [52], Table S1), and (iii) brown spot distribution (a Gosler's index that predicted yolk antibody concentration and was uncorrelated with our spectral measures). A fourth potential candidate may be the digital saturation of brown spots (i.e. the strong correlate of PC1) given its positive 34% and negative 50% correlations with spectral chroma and brightness of brown spots, respectively (but see [41], [42]). We also think that a promising avenue for future work on eggshell coloration lies in analytic approaches combining color and pattern [53], [54].

The partial correlations between the PCs and brown-spotted surface in our study, which specifically avoided spectrometric measurements covering the eggshell ground and spot color simultaneously (unlike [7]), shed some light on the contrasted results obtained previously in blue tits [7], [26]. Indeed, Martínez-de la Puente *et al.* [7] found that more spotted eggs (measured as brown-spotted surface and spectral values covering the eggshell ground and spot color simultaneously) would reflect female physiological stress. In contrast, Sanz & García-Navas [26] and our study (see below) lends support to the opposite prediction, namely that more spotted eggs (measured as PC1 from Gosler's pigmentation indices) may indicate either high female and egg quality (Table S1). The combined results suggest that the two sets of studies probably measured different aspects of brown pigmentation.

While yolk antibody concentration significantly increased with spot size, it is surprising that it did not increase with either the spotted surface, the digital saturation of brown spots, or spectral chroma of brown spots, as they all should reflect protoporphyrin content too. We do not have a satisfying explanation for the absence of a link with the formers, but the relationship with the latter may be masked by sampling variability (measure of different eggs within clutches for yolk compounds and spectral coloration) and/or a lack of statistical power (sample size shrunk by a third). The absence of the relationship between yolk antibody concentration and spectral chroma of brown spots may also be due to a biased sample regarding spot size variation (the spectral probe mount did not allow measuring spot smaller than 2.2 mm in diameter), but we think it is unlikely since a random sampling of the spots with respect to their size with the HSB method on the full egg sample showed no strong correlations between the digital measures of brown spots and spot size (Table 1). In spite of these potential limitations, our finding that spot size and distribution predict yolk antibody concentration nevertheless suggests that both the quantity and the distribution of protoporphyrin pigments in the eggshell reflect the transfer of maternal immunity to the egg yolk.

Many studies of maternally transferred immunity rely on the untold assumption that a higher level of maternally transferred antibodies will increase the fitness of the chick. However, this assumption remains to be tested experimentally in natural conditions [55]. Moreover, a high concentration of total antibodies in egg yolk may reflect either the capacity of the mother to transfer antibodies to the developing embryo or a high level of past maternal infection [19]. In our study, yolk total antibody concentration positively related to several key proxies of maternal quality, i.e. egg volume (a good proxy of chicks' early survival within clutches [56]), the residuals of clutch size on laying date (e.g. [56], [57]), yellow feather brightness of females (e.g. [30], [58]), and their tarsus length (e.g. [59]). This suggests that high-quality mothers were able to transfer higher concentrations of total antibodies to their egg yolks (e.g. as in collared flycatchers [60]). Alternatively, these females might have been more exposed to parasites, which raises the question of an age-specific effect as older females may have been exposed to more parasites across the course of their life. Against this argument, however, we showed that adult and yearling females did not differ in yolk antibody concentration and further analyses showed that yolk antibody concentration did not significantly vary with mother age (1–5 years) in linear and quadratic relationships (all *P*>0.3, *n* = 38).

Either ways, these results suggest in turn that eggshells with larger spots (i.e. more protoporphyrins) concentrated at their broad poles indicate higher egg quality in blue tits. This finding thus supports the first prediction of the SSECH: a large amount of protoporphyrins in eggshells should indicate high egg quality if it reflects the female's high anti-oxidant capacity to withstand the pro-oxidants or to transfer them efficiently to the eggshell [1]. A similar relationship was previously reported in blue tits in which positive correlations were found between brown pigment darkness (PC1) (with loadings quite comparable to ours, Table S1) and two proxies of egg quality, namely eggshell thickness and hatching success [26]. However, our finding also seems to contrast with recent ones in blue tits that suggest a wider distribution of spots for high-quality eggs [23], while we propose that higher egg quality have a restricted distribution of spots at their broad poles. Brown spot size and distribution are likely determined by different mechanisms (physiology and calcium availability, respectively). Thus they may well signal different aspects of female and egg quality depending on the prevailing environmental constraints. In this respect, the study sites in [23] appear to be very poor in calcium as compared to our calcium-rich study site (La Rouvière has calcareous soil). A lack of calcium, which is a critical limiting resource for small birds that are unable to store it for shell formation [61], may strongly constrained egg structure and affect pigment distribution [22]. Therefore, environmental conditions need to be taken into account when studying the signaling function of eggshell spottiness.

In agreement with the lack of correlation between yolk antibody and carotenoid concentrations as well as between male and female phenotypes (no evidence of assortative mating by plumage color, tarsus length, or age; all correlations with *P*≥0.3, *n* = 29, but for age *n* = 25), the parental traits predicting carotenoid concentrations were different from those predicting antibody concentrations. Mothers deposited more carotenoids in their egg yolk when mated with males with brighter blue and yellow feathers. The latter has been reported to signal higher paternal quality in blue tits [58]. Thus the relationship between yolk carotenoids and male feather brightness suggests a positive differential allocation by females. Differences in carotenoid allocation strategies with regard to paternal traits commonly exist in passerines (e.g. compensatory strategy [47], differential allocation [46], no effects [49]). Nevertheless as in the present study, differential allocation of yolk carotenoids (estimated by yolk hue) was previously suggested to occur with males of more pronounced UV coloration of blue feathers (UV contrast) in blue tits [46]. However, given the correlative nature of both studies, it should be recognized that the increase in yolk carotenoids with male plumage brightness might be linked to a higher quality of territory or male courtship feeding (before or during egg laying), alternatives that may be excluded with experimental testing. (Additional discussion concerning yolk carotenoid variation is available in Discussion S1)

To conclude, our study by investigating for the first time the relationships between brown eggshell coloration and egg content showed that eggshell spottiness has the potential to be an intra-specific signal conveying multiple messages. Although correlative, our study should help guide future experimental work aiming at disentangling the various potential (sexual) signals of eggshell spottiness, for instance when coupling adult phenotypic traits manipulation (e.g. attractiveness manipulation, food supplementation) with eggshell color manipulation. Last but not least, our comparison of frequently used estimators of brown eggshell coloration pushed to be more consistent and careful in their utilization.

## Supporting Information

Table S1 **Relationships between indicator traits of female, male, and offspring quality and the different eggshell-color traits measured in studies investigating the potential role of protoporphyrin content in eggshell as a sexual signal.**
(DOC)

Table S2 **Full models of yolk antibody concentration in relation to egg, female, and male traits.**
(DOC)

Table S3 **Full models of yolk carotenoid concentration in relation to egg, female, and male traits.**
(DOC)

Methods S1 **Plumage coloration by spectrometry.**
(DOC)

Results S1 **The predictors of yolk compounds.**
(DOC)

Discussion S1 **Yolk carotenoid variation.**
(DOC)
