# Supplementary material for: Eggshell Spottiness Reflects Maternally Transferred Antibodies in Blue Tits
Source: PLoS One. 2012 Nov 30;7(11):e50389. doi: 10.1371/journal.pone.0050389 (PMC3511563; doi:10.1371/journal.pone.0050389)
Supplement: Table S1 — Relationships between indicator traits of female, male, and offspring quality and the different eggshell-color traits measured in studies investigating the potential role of protoporphyrin content in eggshell as a sexual signal. (DOC) [file pone.0050389.s001.doc]

**Table S1.** Relationships between indicator traits of female, male, and offspring quality and the different eggshell-color traits measured in studies investigating the potential role of protoporphyrin content in eggshell as a sexual signal.

| **Species** | **Eggshell color** | **Nest** | **References** | **Measure of eggshell-color traits** | | **Correlations with indicator traits of quality in** | | | | **Approach** | **Support SSECH?** |
| --- | --- | --- | --- | --- | --- | --- | --- | --- | --- | --- | --- |
|  |  |  |  | **Technique** | **Measured trait** | **Eggs** | **Nestlings** | **Females** | **Males** |  |  |
| Blue tit *Cyanistes caeruleus* | White, speckled with brown spots | In cavity | 3 | Spectrometry (360-740 nm) covering white and brown patches simultaneously^1^ | PC1 (brightness)^2^ |  | n/a | **-: age**; **+: HSP70**; 0: clutch size | **-: IGs**; **+: HSP70**; 0: age, condition | Correlative | Yes but in the opposite direction to all other studies supporting the hypothesis |
|  |  |  |  |  | PC2 (hue)^4^ | +: mass° | n/a | **-: HSP70**; **+: condition**; 0: clutch size | **-: HSP70**; 0: age, condition |  |  |
|  |  |  |  | Computer-analyzed pictures | Brown-spotted surface |  | n/a | **-: condition**, IGs°; 0: clutch size | **-: IGs**; 0: age, condition |  |  |
|  |  |  |  | Computer-analyzed pictures | Brown-spotted surface^5^ | n/a | n/a | n/a | n/a | Correlative | Yes |
|  |  |  |  | Gosler’s pigmentation indices^7^ | Brown pigment darkness (PC1: 47.4%, I = 0.831, D = -0.257, S = 0.815) | **+: eggshell thickness & weight^8^, hatching success** | 0: mass, fledging success | +: **tarsus length**; 0: age, mass, clutch size, incubation behaviour^9^, young feeding effort | 0: age, mass, tarsus length, incubation courtship feeding^10^, young feeding effort |  |  |
|  |  |  |  |  | Brown pigment spread (PC2: 32.8%, I = 0.086, D = 0.963, S = 0.215) | **+: eggshell thickness & weight^8^, daily rate of mass loss during incubation, hatching success** | **+: tarsus length**; 0: mass, fledging success | **+: clutch size,** tarsus length°; 0: age, mass, incubation behaviour^9^, young feeding effort | **+: mass,** **young feeding effort**; 0: age, tarsus length, incubation courtship feeding^10^ |  |  |
|  |  |  | This study | Spectrometry (300-700 nm) | White eggshell brightness, UV chroma and chroma | 0: yolk IGs & carotenoids | n/a | n/a | n/a | Correlative | Yes but little |
|  |  |  |  |  | Brown spot brightness and chroma^5^ | 0: yolk IGs & carotenoids | n/a | n/a | n/a |  |  |
|  |  |  |  | Computer-analyzed pictures | Brown-spotted surface | 0: yolk IGs & carotenoids | n/a | n/a | n/a |  |  |
|  |  |  |  | RGB from pictures | Brown spot hue, saturation and brightness^6^ | 0: yolk IGs & carotenoids | n/a | n/a | n/a |  |  |
|  |  |  |  | Gosler’s pigmentation indices^7^ | Brown pigment darkness (PC1: 65.6%, I = 0.615, D = – 0.584, S = 0.530) | **+: yolk IGs**; 0: yolk carotenoids | n/a | n/a | n/a |  |  |
|  |  |  |  |  | Brown pigment spread (PC2: 21.3%, I = -0.203, D = 0.531, S = 0.822) | 0: yolk IGs & carotenoids | n/a | n/a | n/a |  |  |
| House sparrow *Passer domesticus* | White to bluish, speckled with brown spots | Enclosed or in cavity |  | Gosler’s pigmentation indices^7^ | Brown pigment darkness (PC1: 47.5%, I = 0.780, D = 0.325, S = 0.842) | n/a | n/a | **+: age, clutch size** | n/a | Correlative | Yes |
|  |  |  |  |  | Brown pigment spread (PC2: 33.2%, I = 0.377, D = 0.928, S = 0.009) | n/a | n/a | **+: clutch size** | n/a |  |  |
| House wren *Troglodytes aedon* | White to brown, speckled with brown spots | In cavity |  | Spectrometry (400-700nm)^1^ | PC1 original (brightness, 88.1%)^11^ | **+: mass** | n/a | 0: condition | 0: foster young feeding effort | Correlative & experimental (egg cross-fostering)^12^ | Yes but males do not respond to the potential signal of egg quality and female investment |
|  |  |  |  |  | PC2 original (chroma, 8.5%)^13^ |  | n/a | **+: foster young feeding effort**; 0: condition | 0: foster young feeding effort |  |  |
|  |  |  |  |  | PC3 original (chroma, 2.7%) |  | n/a | 0: condition | 0: foster young feeding effort |  |  |
|  |  |  |  |  | PC1 foster (brightness, 88.1%)^11^ |  | n/a | **+: foster young feeding effort** | 0: foster young feeding effort |  |  |
|  |  |  |  |  | PC2 foster (chroma, 8.5%) ^13^ and PC3 foster (chroma, 2.7%) |  | n/a |  | 0: foster young feeding effort |  |  |
|  |  |  |  | Own pigmentation index^5^ | Bright (white, few spots) to dark (brown, many spots) | n/a | n/a | n/a | n/a |  |  |
| Eurasian kestrel *Falco tinnunculus* | White to dark reddish brown, speckled with brown spots | In cavity |  | RGB from pictures | PC1 (dull/light color, 95.95%)^14^ | +: **mass & hatching success in food-supplemented pairs only**; 0: mass, hatching probability & success | 0: mass, brood reduction, fledging success | +: **age, grey on tail**; 0: mass, tarsus & wing length, condition, brood & clutch size, grey on rump | +: **condition**; 0: mass, tarsus & wing length | Correlative & experimental (food supplementation before egg laying) | Inconclusive as female condition was not affected by treatment |
| Ring-billed gulls *Larus delawarensis* | Brown to olive green or blue green, speckled with brown spots | Open |  | Spectrometry (300-700nm)^15^ | Red eggshell chroma^16^ | 0: mass | **+: mass** | 0: condition, H/L ratio | 0: paternal investment^17^ in relation to original or cross-fostered egg color | Correlative & experimental (egg cross-fostering) | Little |
| Northern lapwing  *Vanellus vanellus* | Beige to brown, speckled with deep-brown to black  Spots | Open |  | Gosler’s pigmentation indices^7,18^ | I | 0: volume, thickness |  | 0: clutch size | 0: incubation | Correlative | No |
|  |  |  |  |  | D | 0: volume |  | 0: clutch size | 0: incubation |  |  |
|  |  |  |  | Computer-analyzed pictures | Brown-spotted surface | 0: volume |  | 0: clutch size | 0: incubation |  |  |
| Great tits *Parus Major* | White, speckled with brown spots | In cavity |  | Computer-analyzed UV-pictures^19^ | PC1 original and (speckling intensity, 49.0%)^19^ | 0: mass | 0: mass | -: **mass**; 0: tarsus length, clutch size, foster young feeding effort^20^ | 0: mass, tarsus length, foster young feeding effort^20^ | Experimental (double cross-fostering at incubation and chick stages) | No |
|  |  |  |  |  | PC1 foster (speckling intensity, 49.0%)^19^ | n/a | 0: mass | n/a | 0: foster young feeding effort^20^ |  |  |

Significant relationships are in bold. When available from literature, we give explained percentages of the total variance and factor loadings of each principal component (PC). SSECH, sexually selected egg color hypothesis ; HSP70, heat shock protein (a stress protein); IGs, immunoglobulins; I, spot intensity; D, spot distribution; S, spot size; RGB, red green blue; H/L ratio, heterophil to lymphocyte ratio in blood (measure of immune stress). n/a, not applicable.

° Marginally significant.

^1^ Composite measure of the eggshell ground and spot color.

^2^ PC1 lower values are for brighter eggs.

^3^ These authors do not consider eggshell coloration as a signal.

^4^ PC2 lower values are for reddish eggs due to spots.

^5^ Compared to other eggshell-color traits within the study.

^6^ From HSB (hue, saturation, brightness) color space.

^7^ See Gosler *et al.* .

^8^ Analysis conducted on unhatched eggs with brown pigment darkness (PC1): 61.8%, I = 0.830, D = 0.151, S = 0.913; and brown pigment spread (PC2): 25.5%, I = 0.316, D = 0.981, S = 0.083.

^9^ Average duration females spent in and out of their nest in 1h of observation during incubation.

^10^ Number of food passes by the male to its female mate at the nest in 1h of observation during incubation.

^11^ PC1 higher values are for brighter eggs.

^12^ Females with brighter foster eggs than their original eggs feed their foster young more than females whose foster eggs were less bright than their original eggs.

^13^ PC2 higher values were interpreted as redder eggs.

^14^ PC1 higher values are associated with highly pigmented eggs. All statistical models included the PC1 (94.9% of total variance) of the grey reference chip placed beside the egg on each picture as covariable.

^15^ Only measured patches of ground coloration (resulting from a combination of protoporphyrin and biliverdin pigments) free of spots.

^16^ Proportion of reflectance in the red portion of the spectrum (300-700 nm).

^17^ Long call rate, feeding rate, neighbor threatening rate, and brooding length.

^18^ The authors did not calculate the PCs from the three Gosler’s pigmentation indices (Intensity I, Distribution D, Size S) and spot size was not used in analyses since spot size and spotted surface (percentage cover of speckling) were strongly correlated (*r* = 0.78, *P* < 0.0001, *n* = 29 clutches), and according to the authors, the spotted surface should describe the characteristics of the eggshell more exactly than spot size which is based on human scoring.

^19^ The four extracted variables (i.e. pattern coverage, pattern dispersion, predominant spot size and pigment darkness) contribute equally to PC1. High values of PC1 correspond to eggs with large, highly contrasting spots that covered a high proportion of the egg and were unevenly dispersed. The authors only gave results on PC1 as statistical analyses performed on PC2 (explaining 26% of the variation) and all four pattern variables independently gave qualitatively similar results to those obtained using PC1.

^20^ Visit rate at the nest and amount of prey delivered to the foster nestlings in 4 hours of observation.

**References**

1. Martínez-de la Puente J, Merino S, Moreno J, Tomas G, Morales J, et al. (2007) Are eggshell spottiness and colour indicators of health and condition in blue tits *Cyanistes caeruleus*? Journal of Avian Biology 38: 377-384.

2. Sanz JJ, García-Navas V (2009) Eggshell pigmentation pattern in relation to breeding performance of blue tits *Cyanistes caeruleus*. Journal of Animal Ecology 78: 31-41.

3. López-de-Hierro MDG, De Neve L (2010) Pigment limitation and female reproductive characteristics influence egg shell spottiness and ground colour variation in the house sparrow (*Passer domesticus*). Journal of Ornithology 151: 833-840.

4. Walters LA, Getty T (2010) Are brighter eggs better? Egg color and parental investment by house wrens. Journal of Field Ornithology 81: 155-166.

5. Martínez-Padilla J, Dixon H, Vergara P, Pérez-Rodríguez L, Fargallo JA (2010) Does egg colouration reflect male condition in birds? Naturwissenschaften 97: 469-477.

6. Hanley D, Doucet SM (2009) Egg coloration in ring-billed gulls (*Larus delawarensis*): a test of the sexual signaling hypothesis. Behavioral Ecology and Sociobiology 63: 719-729.

7. Bulla M, Šálek M, Gosler AG (2012) Eggshell spotting does not predict male incubation but marks thinner areas of a Shorebird's shells. Auk 129: 26-35.

8. Stoddard MC, Fayet AL, Kilner RM, Hinde CA (2012) Egg speckling patterns do not advertise offspring quality or influence male provisioning in great tits. Plos one 7.

9. Moreno J, Osorno JL (2003) Avian egg colour and sexual selection: does eggshell pigmentation reflect female condition and genetic quality? Ecology Letters 6: 803-806.

10. Gosler AG, Barnett PR, Reynolds SJ (2000) Inheritance and variation in eggshell patterning in the great tit *Parus major*. Proceedings of the Royal Society of London Series B-Biological Sciences 267: 2469-2473.

11. Gosler AG, Higham JP, Reynolds SJ (2005) Why are birds' eggs speckled? Ecology Letters 8: 1105-1113.
