# Supplementary material for: Eggshell Spottiness Reflects Maternally Transferred Antibodies in Blue Tits
Source: PLoS One. 2012 Nov 30;7(11):e50389. doi: 10.1371/journal.pone.0050389 (PMC3511563; doi:10.1371/journal.pone.0050389)
Supplement: Table S2 — Full models of yolk antibody concentration in relation to egg, female, and male traits. (DOC) [file pone.0050389.s002.doc]

**Table S2.** Full models of yolk antibody concentration in relation to egg, female, and male traits.

| **Model terms** | **Estimate ± 1 SE** | ***F*** | **d.f.** | ***P*** |
| --- | --- | --- | --- | --- |
| **Egg and female traits**^1^ |  |  |  |  |
| *Pigment darkness (PC1)* | *0.042 ± 0.012* | *12.1* | *1,23* | *0.002* |
| *Tarsus length* | *0.140 ± 0.061* | *5.3* | *1,23,23* | *0.03* |
| Laying order° | -0.016 ± 0.008 | 3.8 | 1,23 | 0.06 |
| *Residuals of clutch size on laying date*^§^ | *0.027 ± 0.014* | *3.7* | *1,23,23* | *0.07* |
| *Yellow feather brightness*^§^ | *0.019 ± 0.010* | *3.6* | *1,23,23* | *0.07* |
| Laying date° | 0.009 ± 0.005 | 3.4 | 1,23,23 | 0.08 |
| Yellow feather chroma | -0.192 ± 0.133 | 2.1 | 1,23,23 | 0.2 |
| *Egg volume** | *0.225 ± 0.189* | *1.4* | *1,23* | *0.2* |
| Blue feather hue | -0.001 ± 0.001 | 1.0 | 1,23,23 | 0.3 |
| Blue feather brightness | -0.005 ± 0.005 | 1.0 | 1,23,23 | 0.3 |
| Age | -0.037 ± 0.040 | 0.9 | 1,23,23 | 0.4 |
| Brown-spotted surface | -0.342 ± 0.456 | 0.6 | 1,23 | 0.5 |
| Digital brightness of brown spots | 0.159 ± 0.213 | 0.6 | 1,23 | 0.5 |
| Pigment spread (PC2) | 0.009 ± 0.017 | 0.3 | 1,23 | 0.6 |
| Digital hue of brown spots | 0.039 ± 0.080 | 0.2 | 1,23 | 0.6 |
| Spectral UV chroma of white eggshell | 0.754 ± 2.651 | 0.1 | 1,23 | 0.8 |
| Spectral brightness of white eggshell | 0.0003 ± 0.002 | 0.03 | 1,23 | 0.9 |
| Digital saturation of white eggshell | -0.075 ± 0.441 | 0.03 | 1,23 | 0.9 |
| Days of clutch incubation | -0.001 ± 0.019 | <0.01 | 1,23,23 | 0.95 |
| Spectral chroma of white eggshell | -0.002 ± 0.418 | <0.01 | 1,23 | 0.99 |
| **Male traits**^2^ |  |  |  |  |
| *Egg volume* | *0.468 ± 0.155* | *9.2* | *1,21* | *0.006* |
| *Residuals of clutch size on laying date* | *0.071 ± 0.033* | *4.5* | *1,13,21* | *0.05* |
| Yellow feather brightness | -0.036 ± 0.021 | 3.0 | 1,13,21 | 0.1 |
| Tarsus length | 0.200 ± 0.131 | 2.3 | 1,13,21 | 0.2 |
| Laying date | 0.011 ± 0.008 | 1.7 | 1,13,21 | 0.2 |
| Laying order | -0.007 ± 0.005 | 1.5 | 1,21 | 0.2 |
| Days of clutch incubation | -0.048 ± 0.040 | 1.5 | 1,13,21 | 0.2 |
| Age | -0.030 ± 0.093 | 0.1 | 1,13,21 | 0.8 |
| Yellow feather chroma | -0.058 ± 0.321 | 0.03 | 1,13,21 | 0.9 |
| Blue feather brightness | 0.002 ± 0.009 | 0.04 | 1,13,21 | 0.8 |
| Blue feather hue | 0.0005 ± 0.005 | 0.01 | 1,13,21 | 0.9 |

Separate mixed model analyses were performed for egg and female traits and male traits. The traits found to be significant (or close to significance) in minimal models (Table 2) are in italics.

^1^ 67 eggs from 33 clutches.

^2^ 46 eggs from 23 clutches.

° These traits were not retained in the minimal model (Table 2). However, the fit of the model was improved (but not significantly) when keeping them in it (AIC with them =-125.8, without = -124.9).

^§^ These traits were retained in the minimal model (Table 2) while they do not have a significant effect on yolk antibody concentration in simple models (i.e. with one explanatory factor; residuals of clutch size on laying date: Estimate ± 1 SE = 0.025 ± 0.016, *F*_1,31,34_ = 2.3, *P* = 0.1; yellow feather brightness: 0.018 ± 0.011, *F*_1,31,34_ = 2.6, *P* = 0.1). Nevertheless, note that the effect sizes are highly similar among the various models (residuals of clutch size on laying date, simple model: 0.025 ± 0.016; full model: 0.027 ± 0.014, this table, minimal model: 0.029 ± 0.013, Table 2; yellow feather brightness, simple model: 0.018 ± 0.011; full model: 0.018 ± 0.010, this table, minimal model: 0.023 ± 0.009, Table 2). In addition, clutch size residuals had a significant effect on yolk antibody concentration within the egg- and female-trait database (0.029 ± 0.013, *F*_1,29,32_ = 5.2, *P* = 0.03) and within the male-trait database (0.044 ± 0.022, *F*_1,21,22_ = 4.2, *P* = 0.05), making us confident that this variable is not a false positive . Yellow feather brightness is thus the only parameter that we spotted as a potential false positive.

* This trait was retained in the minimal model (Table 2), but is likely not a false positive given its effect on yolk antibody concentration in the simple models (i.e. with one explanatory factor) within the egg- and female-trait database (Estimate ± 1 SE = 0.260 ± 0.134, *F*_1,33_ = 3.7, *P* = 0.06) and within the male-trait database (0.387 ± 0.137, *F*_1,22_ = 8.0, *P* = 0.01).

**References**

1. Forstmeier W, Schielzeth H (2011) Cryptic multiple hypotheses testing in linear models: Overestimated effect sizes and the winner's curse. Behavioral Ecology and Sociobiology 65: 47-55.
