# Supplementary material for: Eggshell Spottiness Reflects Maternally Transferred Antibodies in Blue Tits
Source: PLoS One. 2012 Nov 30;7(11):e50389. doi: 10.1371/journal.pone.0050389 (PMC3511563; doi:10.1371/journal.pone.0050389)
Supplement: Table S3 — Full models of yolk carotenoid concentration in relation to egg, female, and male traits. (DOC) [file pone.0050389.s003.doc]

**Table S3.** Full models of yolk carotenoid concentration in relation to egg, female, and male traits.

| **Model terms** | **Estimate ± 1 SE** | ***F*** | **d.f.** | ***P*** |
| --- | --- | --- | --- | --- |
| **Egg and female traits**^1^ |  |  |  |  |
| Pigment darkness (PC1)° | -0.097 ± 0.056 | 3.0 | 1,22 | 0.1 |
| *Days of clutch incubation** | *-0.114 ± 0.083* | *1.9* | *1,23,23* | *0.2* |
| *Laying order** | *-0.043 ± 0.038* | *1.3* | *1,22* | *0.3* |
| Digital saturation of white eggshell | 2.339 ± 2.071 | 1.3 | 1,22 | 0.3 |
| Laying date | -0.023 ± 0.021 | 1.2 | 1,23,23 | 0.3 |
| Age | 0.171 ± 0.175 | 1.0 | 1,23,23 | 0.3 |
| Yellow feather brightness | 0.038 ± 0.045 | 0.7 | 1,23,23 | 0.4 |
| Blue feather brightness | -0.014 ± 0.022 | 0.4 | 1,23,23 | 0.5 |
| Yellow feather chroma | -0.390 ± 0.595 | 0.4 | 1,23,23 | 0.5 |
| Egg volume | 0.505 ± 0.865 | 0.3 | 1,22 | 0.6 |
| Spectral UV chroma of white eggshell | -6.125 ± 12.314 | 0.2 | 1,22 | 0.6 |
| Blue feather hue | 0.003 ± 0.006 | 0.2 | 1,23,23 | 0.6 |
| Digital brightness of brown spots | 0.427 ± 0.989 | 0.2 | 1,22 | 0.7 |
| Digital hue of brown spots | -0.151 ± 0.374 | 0.2 | 1,22 | 0.7 |
| Pigment spread (PC2) | -0.022 ± 0.079 | 0.1 | 1,22 | 0.8 |
| Residuals of clutch size on laying date | 0.017 ± 0.063 | 0.1 | 1,23,23 | 0.8 |
| Brown-spotted surface | 0.511 ± 2.116 | 0.1 | 1,22 | 0.8 |
| Spectral brightness of white eggshell | -0.001 ± 0.007 | 0.04 | 1,22 | 0.9 |
| Tarsus length | -0.035 ± 0.271 | 0.02 | 1,23,23 | 0.9 |
| Spectral chroma of white eggshell | 0.231 ± 1.905 | 0.1 | 1,22 | 0.9 |
| **Male traits**^2^ |  |  |  |  |
| *Blue feather brightness* | *0.066 ± 0.019* | *11.5* | *1,13,20* | *0.005* |
| Blue feather hue° | -0.022 ± 0.011 | 4.6 | 1,13,20 | 0.05 |
| *Yellow feather brightness* | *0.105 ± 0.049* | *4.5* | *1,13,20* | *0.05* |
| *Laying order** | *-0.054 ± 0.028* | *3.6* | *1,20* | *0.07* |
| *Days of clutch incubation** | *-0.158 ± 0.089* | *3.2* | *1,13,20* | *0.09* |
| Residuals of clutch size on laying date | 0.101 ± 0.076 | 1.8 | 1,13,20 | 0.2 |
| Laying date | -0.021 ± 0.018 | 1.4 | 1,13,20 | 0.3 |
| Age | 0.158 ± 0.211 | 0.6 | 1,13,20 | 0.5 |
| Egg volume | -0.294 ± 0.647 | 0.2 | 1,20 | 0.7 |
| Yellow feather chroma | -0.211 ± 0.716 | 0.1 | 1,13,20 | 0.8 |
| Tarsus length | -0.025 ± 0.292 | 0.01 | 1,13,20 | 0.9 |

Separate mixed model analyses were performed for egg and female traits and male traits. The traits found to be significant (or close to significance) in minimal models (Table 3) are in italics.

^1^ 66 eggs from 33 clutches.

^2^ 45 eggs from 23 clutches.

° These traits were not retained in the minimal model (Tables 2, 3). However, the fit of the models was improved (but not significantly) when keeping the traits in them (PC1: AIC with the trait = 66.2, without = 66.6; blue feather hue: AIC with the trait = 31.7, without = 32.1).

* These traits were retained in the minimal models (Table 3), but are likely not false positives given their effects on yolk antibody concentration in the simple models (i.e. with one explanatory factor) within the egg- and female-trait database (laying order: Estimate ± 1 SE = -0.084 ± 0.025, *F*_1,32_ = 11.6, *P* = 0.002; days of clutch incubation: -0.143 ± 0.058, *F*_1,31,33_ = 6.1, *P* = 0.02) and within the male-trait database (laying order: -0.053 ± 0.025, *F*_1,21_ = 4.4, *P* = 0.05; days of clutch incubation: -0.264 ± 0.097, *F*_1,21,22_ = 7.4, *P* = 0.01).

**References**

1. Forstmeier W, Schielzeth H (2011) Cryptic multiple hypotheses testing in linear models: Overestimated effect sizes and the winner's curse. Behavioral Ecology and Sociobiology 65: 47-55.
