# Supplementary material for: Eggshell Spottiness Reflects Maternally Transferred Antibodies in Blue Tits
Source: PLoS One. 2012 Nov 30;7(11):e50389. doi: 10.1371/journal.pone.0050389 (PMC3511563; doi:10.1371/journal.pone.0050389)
Supplement: Methods S1 — Plumage coloration by spectrometry. (DOC) [file pone.0050389.s004.doc]

**Methods S1.** Plumage coloration by spectrometry.

Following bird capture, we collected six feathers of the UV-blue cap and eight of the yellow collar per bird. Color was measured as described in [1] with an AVASPEC-2048 spectrometer (Avantes, NL) with a deuterium-halogen light source (AVALIGHT-DH-S lamp, Avantes, NL) covering the range 300–700 nm and a 200-lm fibre optic probe. The procedure was identical to the one described for eggshell measurements. For each bird and body part (collar or cap), we computed the mean of six reflectance spectra taken from two sets of three and four feathers for blue and yellow coloration, respectively.

Following a previous study on the same blue tit population [1], we computed blue brightness and hue, and yellow brightness and chroma. For both color patches, brightness was the mean reflectance over the range 300-700 nm (e.g. [2,3]). Hue and chroma were computed based on the shape of reflectance spectra (e.g. [4,5,6,7]). For UV-blue coloration, we computed hue as the wavelength at maximal reflectance over the range 300-700 nm. For yellow coloration, we computed chroma as (R_700_-R_450_) ⁄ (R average _300-700_) as this should directly indicate the influence of carotenoids, which maximally absorb around 450 nm [8]. These measurements were significantly repeatable (blue: 0.55 > *R* > 0.76, yellow: 0.42 > *R* > 0.80, all *F*_69,350_ > 5.4, *P* < 0.001). In both sexes, blue brightness was not correlated with blue hue (males: Spearman *rho*_27_ = 0.18, females: Pearson *r*_39_ = 0.24, both *P* > 0.12) and yellow brightness was not correlated with yellow chroma (males: *r*_27_ = -0.10, females: *r*_39_ = -0.09, both *P* > 0.56).

**References**

1. Doutrelant C, Grégoire A, Grnac N, Gomez D, Lambrechts MM, et al. (2008) Female coloration indicates female reproductive capacity in blue tits. Journal of Evolutionary Biology 21: 226-233.

2. Delhey K, Peters A, Johnsen A, Kempenaers B (2006) Seasonal changes in blue tit crown color: do they signal individual quality? Behavioral Ecology 17: 790-798.

3. Andersson S, Prager M, Johansson EIA (2007) Carotenoid content and reflectance of yellow and red nuptial plumages in widowbirds (*Euplectes* spp.). Functional Ecology 21: 272-281.

4. Andersson S, Ornborg J, Andersson M (1998) Ultraviolet sexual dimorphism and assortative mating in blue tits. Proceedings of the Royal Society of London Series B-Biological Sciences 265: 445-450.

5. Korsten P, Lessells CM, Mateman AC, van der Velde M, Komdeur J (2006) Primary sex ratio adjustment to experimentally reduced male UV attractiveness in blue tits. Behavioral Ecology 17: 539-546.

6. Delhey K, Johnsen A, Peters A, Andersson S, Kempenaers B (2003) Paternity analysis reveals opposing selection pressures on crown coloration in the blue tit (*Parus caeruleus*). Proceedings of the Royal Society of London Series B-Biological Sciences 270: 2057-2063.

7. Griffith SC, Ornborg J, Russell AF, Andersson S, Sheldon BC (2003) Correlations between ultraviolet coloration, overwinter survival and offspring sex ratio in the blue tit. Journal of Evolutionary Biology 16: 1045-1054.

8. Andersson S, Pryke SR, Ornborg J, Lawes MJ, Andersson M (2002) Multiple receivers, multiple ornaments, and a trade-off between agonistic and epigamic signaling in a widowbird. American Naturalist 160: 683-691.
