# Supplementary material for: Eggshell Spottiness Reflects Maternally Transferred Antibodies in Blue Tits
Source: PLoS One. 2012 Nov 30;7(11):e50389. doi: 10.1371/journal.pone.0050389 (PMC3511563; doi:10.1371/journal.pone.0050389)
Supplement: Results S1 — The predictors of yolk compounds. (DOC) [file pone.0050389.s005.doc]

**Results S1.** The predictors of yolk compounds.

The predictors of yolk antibody level

Pigment darkness (PC1) strongly correlated with digital saturation of brown spots (Pearson *r*_65_ = 0.81, *P* < 0.001 within this dataset; see also Table 1). Despite this strong correlation, only PC1 was a good predictor of yolk antibody level. Indeed, when we included both parameters in the full model “egg and female traits”, PC1, but not digital saturation of brown spots, was significant in the full model, but both were retained in the minimal model, no matters whether brown-spotted surface (a strong correlate of PC1, *r*_65_ = 0.65, *P* < 0.001, within this dataset; Table 1) was included (full model: *F*_1,22_ = 12.6 and 1.6, *P* = 0.002 and 0.2, for PC1 and digital saturation of brown spots, respectively - minimal model: *F*_1,31_ = 10.4 and 4.6, *P* = 0.003 and 0.04, for PC1 and digital saturation of brown spots, respectively) or not (full model: *F*_1,23_ = 12.9 and 2.1, *P* = 0.002 and 0.2, for PC1 and digital saturation of brown spots, respectively - minimal model: same as above). In addition, in models including only one or the other of the two highly correlated parameters (i.e. PC1 and digital saturation of brown spots), PC1 was significant in the full and minimal models while digital saturation of brown spots was neither significant in the full nor in the minimal model, again independently of having or not brown-spotted surface in models (for PC1, full model including brown-spotted surface and excluding digital saturation of brown spots: *F*_1,23_ = 12.1, *P* = 0.002, Table S2; full model excluding brown-spotted surface and digital saturation of brown spots: *F*_1,24_ = 11.9, *P* = 0.002; both minimal models: *F*_1,32_ = 5.6, *P* = 0.02, Table 2 - for digital saturation of brown spots, full model including brown-spotted surface and excluding PC1: *F*_1,23_ = 0.8, *P* = 0.4; full model excluding brown-spotted surface and PC1: *F*_1,24_ = 1.1, *P* = 0.3; in both models, rejected at *F*_1,32_ = 0.6, *P* = 0.4). Thus, digital saturation of brown spots appears as a false positive and the presence or absence of brown-spotted surface in models does not change the outcome. Therefore in the manuscript, we present the results of the model that excludes digital saturation of brown spots but includes brown-spotted surface (Tables 2, S2). Note that in all the mentioned minimal models, in addition to PC1, we always found the following predictors of yolk antibody level, i.e. effects of the residuals of clutch size on laying date, egg volume, yellow feather brightness, and tarsus length of females (statistics not shown).

The predictors of yolk carotenoid level

As above, we also checked the potential influence of the high correlations between parameters on the set of good predictors of yolk carotenoid level. All models led to the same outcome, even when including digital saturation of brown spots (statistics not shown), but for consistency, we also present the results of the model “egg and female traits” that excludes digital saturation of brown spots (Table S3).

**References**

1. Forstmeier W, Schielzeth H (2011) Cryptic multiple hypotheses testing in linear models: Overestimated effect sizes and the winner's curse. Behavioral Ecology and Sociobiology 65: 47-55.
