# Supplementary material for: Eggshell Spottiness Reflects Maternally Transferred Antibodies in Blue Tits
Source: PLoS One. 2012 Nov 30;7(11):e50389. doi: 10.1371/journal.pone.0050389 (PMC3511563; doi:10.1371/journal.pone.0050389)
Supplement: Discussion S1 — Yolk carotenoid variation. (DOC) [file pone.0050389.s006.doc]

**Discussion S1.** Yolk carotenoid variation.

We report a yolk total carotenoid concentration in 2007 similar to the one found in the same population in 2008 (Midamegbe *et al.*, submitted). The concentration in our study population is on average 1.5 times higher than in another population of blue tits breeding in eastern France, but is similar to the concentration found in this population after the females got carotenoid supplementation in diet . Because environmental variability and/or differences in foraging ability, absorption, or metabolizing efficiency (reviewed in ) may make carotenoids a limited resource, this result suggests that carotenoids might be quite abundant in our study population or that females face a lower level of exposure to parasites (that likely limit maternal transfer to the egg yolk ) or stress. Carotenoids can only be acquired from specific food items and play crucial roles in immune defense mechanisms and embryonic development . Thus if they are highly available, female quality should have a limited influence (in terms of foraging and/or immunity capacity) on their allocation to the eggs. In accordance with this idea, none of the several proxies of female quality used in this study predicted egg yolk concentrations. In addition, if females in our population are indeed not limited in carotenoids, the association between yolk carotenoid content and male plumage coloration may be best explained by variation in territory quality rather than differential allocation.

As previously found in several passerines (e.g. ), our results showed a decrease in maternal deposition of yolk carotenoids over the laying sequence (note that the yolk antibody level did not vary with laying order, which is consistent with the absence of systematic variation within clutches ). Carotenoid supplementation in the diets of several bird species did not prevent its decrease over the laying sequence (e.g. , but see ). Moreover, carotenoids might not be limited in our study population. It suggests that there might be a trade-off with mother’s own somatic maintenance to fight oxidative stress accumulated over the egg laying or an active regulation of antioxidant concentration in eggs by females (control over sibling competition, offspring sex or brood reduction, e.g. ; differential allocation in favor of extra-pair young ).

**References**

1. Biard C, Surai PF, Møller AP (2005) Effects of carotenoid availability during laying on reproduction in the blue tit. Oecologia 144: 32-44.

2. Olson VA, Owens IPF (1998) Costly sexual signals: are carotenoids rare, risky or required? Trends in Ecology & Evolution 13: 510-514.

3. Møller AP, Biard C, Blount JD, Houston DC, Ninni P, et al. (2000) Carotenoid-dependent signals: indicators of foraging efficiency, immunocompetence or detoxification ability? Avian and Poultry Biology Reviews 11: 137-159.

4. Saino N, Bertacche V, Ferrari RP, Martinelli R, Møller AP, et al. (2002) Carotenoid concentration in barn swallow eggs is influenced by laying order, maternal infection and paternal ornamentation. Proceedings of the Royal Society of London Series B-Biological Sciences 269: 1729-1733.

5. Goodwin TW (1984) The biochemistry of carotenoids. London: Chapman & Hall.

6. Costantini D, Møller AP (2008) Carotenoids are minor antioxidants for birds. Functional Ecology 22: 367-370.

7. Surai PF, Speake BK, Sparks NHC (2001) Carotenoids in avian nutrition and embryonic development. 2. Antioxidant properties and discrimination in embryonic tissues. Journal of Poultry Science 38: 117-145.

8. Hõrak P, Surai PF, Møller AP (2002) Fat-soluble antioxidants in the eggs of great tits Parus major in relation to breeding habitat and laying sequence. Avian Science 2: 123-130.

9. Cassey P, Ewen JG, Boulton RL, Blackburn TM, Møller AP, et al. (2005) Egg carotenoids in passerine birds introduced to New Zealand: relations to ecological factors, integument coloration and phylogeny. Functional Ecology 19: 719-726.

10. Royle NJ, Surai PF, Hartley IR (2003) The effect of variation in dietary intake on maternal deposition of antioxidants in zebra finch eggs. Functional Ecology 17: 472-481.

11. Newbrey JL, Reed WL, Foster SP, Zander GL (2008) Laying-sequence variation in yolk carotenoid concentrations in eggs of Yellow-headed Blackbirds (*Xanthocephalus xanthocephalus*). Auk 125: 124-130.

12. Saino N, Romano M, Ferrari RP, Martinelli R, Møller AP (2003) Maternal antibodies but not carotenoids in barn swallow eggs covary with embryo sex. Journal of Evolutionary Biology 16: 516-522.

13. Boulinier T, Staszewski V (2008) Maternal transfer of antibodies: raising immuno-ecology issues. Trends in Ecology & Evolution 23: 282-288.

14. Blount JD, Surai PF, Nager RG, Houston DC, Møller AP, et al. (2002) Carotenoids and egg quality in the lesser black-backed gull *Larus fuscus*: a supplemental feeding study of maternal effects. Proceedings of the Royal Society of London Series B-Biological Sciences 269: 29-36.

15. von Schantz T, Bensch S, Grahn M, Hasselquist D, Wittzell H (1999) Good genes, oxidative stress and condition-dependent sexual signals. Proceedings of the Royal Society of London Series B-Biological Sciences 266: 1-12.

16. Cichon M, Dubiec A, Stoczko M (2003) Laying order and offspring sex in blue tits *Parus caeruleus*. Journal of Avian Biology 34: 355-359.

17. Stenning MJ (2008) Hatching asynchrony and brood reduction in blue tits *Cyanistes caeruleus* may be a plastic response to local oak *Quercus robur* bud burst and caterpillar emergence. Acta Ornithologica 43: 97-106.

18. Berthouly A, Helfenstein F, Tanner M, Richner H (2008) Sex-related effects of maternal egg investment on offspring in relation to carotenoid availability in the great tit. Journal of Animal Ecology 77: 74-82.

19. Magrath MJL, Vedder O, van der Velde M, Komdeur J (2009) Maternal effects contribute to the superior performance of extra-pair offspring. Current Biology 19: 792-797.
